# *Elizabethkingia* Species as an Emerging Pathogen: A Comprehensive Review of Clinical and Microbiological Evidence

**DOI:** 10.3390/pathogens15030278

**Published:** 2026-03-04

**Authors:** Jacqueline Wan Yu Tan, Bernice Jia Xin Lian, Cheryl Ying Xuan Loh, Kay Choong See

**Affiliations:** 1 Yong Loo Lin School of Medicine, National University of Singapore, Singapore 119228, Singapore; e0974484@u.nus.edu (B.J.X.L.); cheryllohyx@u.nus.edu (C.Y.X.L.); 2 Division of Respiratory and Critical Care Medicine, Department of Medicine, National University Hospital, Singapore 119074, Singapore; kaychoongsee@nus.edu.sg

**Keywords:** *Elizabethkingia*, *Elizabethkingia anophelis*, *Elizabethkingia meningoseptica*, neonatal meningitis, healthcare-associated infection, outbreak investigation, antimicrobial resistance, MALDI-TOF MS, whole-genome sequencing, biofilm

## Abstract

*Elizabethkingia* species are rare but increasingly recognised Gram-negative pathogens linked to healthcare-associated transmission, intrinsic multidrug resistance, and severe infection in vulnerable hosts. We performed a comprehensive review of human *Elizabethkingia* infections by systematically searching PubMed on 18 October 2025 and included English-language case reports, case series, and outbreak investigations; species were analysed as reported (legacy nomenclature retained), and adults were defined as ≥18 years. In total, 374 studies were included (300 case reports, 41 case series, 33 outbreak investigations). Adult infections were predominantly healthcare-related, affected older adults with substantial comorbidities and most often presented as bacteraemia or sepsis and pneumonia; crude mortality in adult case reports was 32.8%. Paediatric disease was concentrated in neonates and Neonatal Intensive Care Unit (NICU) settings, with meningitis and bloodstream infection predominating; crude mortality in paediatric case reports was 23.3%, and neurological sequelae were frequently reported among survivors. Across studies, isolates showed broad resistance to β-lactams and near-universal resistance to carbapenems, with variable activity to fluoroquinolones and trimethoprim–sulfamethoxazole and more consistent in vitro activity to minocycline. Species misidentification (notably *Elizabethkingia anophelis* as *Elizabethkingia meningoseptica*) and heterogeneous susceptibility testing limited comparability. Outbreak investigations repeatedly implicated water-associated reservoirs and reusable equipment, underscoring the need for improved diagnostics, susceptibility-guided therapy and water-focused infection prevention.

## 1. Introduction

First described by King in 1959, *Elizabethkingia* species have emerged as a significant pathogen in healthcare settings, causing severe infections predominantly among neonates, immunocompromised patients and critically ill patients [1,2,3,4,5]. These organisms are widely distributed in soil and water [6], and outbreaks have been documented in neonatal and adult intensive care units, often linked to contaminated hospital equipment and environmental reservoirs, including respiratory support devices, ventilation circuits, tap water, sink drains, disinfectants and other liquid solutions [7,8,9,10]. *Elizabethkingia* spp. can persist in moist environments and on medical devices through biofilm formation, facilitating transmission within healthcare settings and limiting the effectiveness of conventional disinfection measures [11,12].

Although *Elizabethkingia* infections remain uncommon in immunocompetent individuals, they are associated with high mortality and morbidity among neonates [13,14,15] and immunocompromised patients. Clinical manifestations most commonly include pneumonia [16,17], meningitis [18,19] and sepsis [20,21], with less frequent involvement of the urinary tract [22,23], eye [24,25] and joints [26,27].

The clinical challenge posed by *Elizabethkingia* spp. is compounded by intrinsic multidrug resistance and persistent difficulties in accurate species-level identification, which hinder timely diagnosis and contribute to poor outcomes. Huang et al. demonstrated that *Elizabethkingia meningoseptica* has mortality rates comparable to non-fermenting Gram-negative bacilli, a group of nosocomial pathogens that are difficult to treat and eradicate [4,28].

*Elizabethkingia* spp. are Gram-negative, non-motile, non-spore-forming aerobic rods [2], first described by Elizabeth O. King in the 1950s as causative agents of infant meningitis and sepsis [6]. Following identification as a cause of infant sepsis and meningitis, the bacterium was initially phenotypically classified as CDC group IIa by the US Center for Disease Control and Prevention (CDC) [29] and was later named *Flavobacterium meningosepticum* by King in 1959 [30], before being reclassified in the genus *Chryseobacterium* by Vandamme et al. in 1994 [31].

In 2003, *Chryseobacterium miricola* was isolated from condensation water on the Russian space station Mir [32]. Subsequent phylogenetic and phenotypic analyses by Kim et al. led to the transfer of *Chryseobacterium meningosepticum* and *Chryseobacterium miricola* into a new genus, *Elizabethkingia*, in 2005 [33].

*Elizabethkingia anophelis* was later identified in 2011 from the midgut of Anopheles gambiae mosquitoes [34]. *Elizabethkingia endophytica*, isolated from sweet corn and described as a novel species [34], was then shown through genomic analyses to be a strain of *E. anopheles* [35].

At present, *Elizabethkingia* comprises eight recognised species, of which three are most commonly implicated in human infection: *Elizabethkingia meningoseptica*, *Elizabethkingia anophelis* and *Elizabethkingia miricola* [29]. Historically, *Elizabethkingia meningoseptica* accounted for the majority of reported human infections [36]. However, earlier reliance on conventional phenotypic identification methods, including MALDI-TOF MS platforms with incomplete reference databases, frequently resulted in species-level misidentification, most notably misclassification of *E. anophelis* as *E. meningoseptica* [37,38,39].

With advances in diagnostic identification methods, the apparent distribution of clinically relevant *Elizabethkingia* species has evolved [38,40]. In a study of 334 clinical isolates collected between 2005 and 2020, *E. anophelis* was identified as the predominant species (86.2%, 288/334), followed by *E. meningoseptica* (8.7%, 29/334), *E. miricola* (4.8%, 16/334) and unclassified *Elizabethkingia* spp. (0.3%, 1/334). These findings highlight the substantial impact of molecular and mass spectrometry-based identification methods on the contemporary understanding of *Elizabethkingia* epidemiology [41].

Despite increasing recognition, available evidence remains fragmented, largely derived from case reports, small case series and outbreak investigations, with substantial heterogeneity in diagnostic methods, antimicrobial susceptibility testing and outcome reporting. Key areas of ongoing uncertainty include the relative contribution of individual *Elizabethkingia* species to human disease, historical misclassification between *Elizabethkingia meningoseptica* and *Elizabethkingia anophelis* and optimal antimicrobial therapy.

This review aims to synthesise current case reports, case series and outbreak investigations to provide a consolidated analysis of *Elizabethkingia* microbiology, pathogenic potential, epidemiology, clinical characteristics and management across adult and paediatric populations. Overall, the evidence indicates that *Elizabethkingia* spp. are predominantly healthcare-associated pathogens characterised by substantial mortality, marked age-specific disease patterns, extensive intrinsic antimicrobial resistance and frequent links to water-associated environmental reservoirs, emphasising the need for improved diagnostic accuracy, susceptibility-guided therapy and effective infection control strategies.

## 2. Microbiology of *Elizabethkingia* spp.

### 2.1. Taxonomy and Classification

*Elizabethkingia* spp. are non-fermenting, aerobic, Gram-negative bacilli belonging to the family Weeksellaceae, previously classified under the genera Flavobacterium and Chryseobacterium [42]. They are non-motile, non-spore-forming organisms that are widely distributed in environmental water and soil [43].

### 2.2. Morphology and Growth Characteristics

On Gram stain, *Elizabethkingia* spp. appear as slender Gram-negative rods and may demonstrate variable staining due to their lipid-rich outer membrane. [12]. They are oxidase-positive and catalase-positive and characteristically non-glucose-fermenting [44]. Growth occurs on routine laboratory media, including blood agar and chocolate agar, producing smooth, pale-yellow to cream-coloured colonies after 24–48 h of incubation at 35–37 °C [45].

*Elizabethkingia* spp. are typically non-lactose fermenters on MacConkey agar and may exhibit weak or absent growth. They are intrinsically resistant to many commonly used antimicrobial agents for Gram-negative infections, a feature that complicates both empirical treatment and laboratory susceptibility interpretation.

### 2.3. Biochemical and Phenotypic Features

*Elizabethkingia* spp. are oxidase-positive and catalase-positive, a key feature that helps differentiate them from other non-fermenting Gram-negative bacilli. They are non-glucose-fermenting, exhibiting oxidative rather than fermentative metabolism, and are indole-positive, a characteristic that aids in distinction from other members of the *Flavobacteriaceae*. Urease and nitrate reduction tests are typically negative.

The intrinsic resistance phenotype is of major clinical significance as *Elizabethkingia* species, particularly *Elizabethkingia meningoseptica*, commonly have three chromosomal β-lactamase genes, including a class A extended-spectrum beta-lactamase, and two metallo-β-lactamases (BlaB and GOB), which break down β-lactams and carbapenems [46,47,48,49]. Meanwhile, *Elizabethkingia anophelis* possess metallo-β-lactamases (BlaB and GOB), which are responsible for carbapenem resistance, and a serine β-lactamase, which is involved in resistance against cephalosporins and monobactams [50,51].

### 2.4. Virulence Factors and Environmental Persistence

Beyond antimicrobial resistance, *Elizabethkingia* spp. exhibits a strong intrinsic capacity for biofilm formation [52]. Multiple factors can promote its biofilm formation, including exposure to moist, nutrient-limited environments, such as sinks, taps and humidifiers [8,53], which causes *Elizabethkingia* spp. to shift from planktonic growth to a biofilm lifestyle [54]. Biofilm can develop on abiotic surfaces, such as plastics and rubber, and healthcare equipment, such as catheters and tubing [9,11]. Biofilm formation on indwelling medical devices and hospital surfaces is believed to facilitate environmental persistence, nosocomial transmission and reduced antimicrobial penetration, contributing to antibiotic resistance [54], thereby contributing to treatment failure and recurrent infection and rendering eradication challenging [12,55].

*Elizabethkingia’s* intrinsic genetic determinants can also collectively facilitate persistence of *Elizabethkingia* in healthcare settings, making it extremely difficult to eradicate and treat [56].

Some *Elizabethkingia* strains are also known to encode genes for proteolytic and tissue-damaging enzymes [55]. These enzymes include metalloproteases, serine proteases, lipases and phospholipases [2], and they may contribute to tissue damage.

*Elizabethkingia* spp. also produces outer membrane vesicles (OMVs), which carry virulence components such as Rag/Sus family proteins, HmuY heme-binding proteins [57], metaloproteases and transporters—all of which facilitate horizontal gene transfer, promote interbacterial communication and enhance survival under antimicrobial pressure. Hence, it greatly contributes to the environmental persistence and severe invasive disease in susceptible patients [57,58].

### 2.5. Antimicrobial Resistance Mechanisms

In addition, *Elizabethkingia* spp. have been shown to develop variable fluoroquinolone resistance during therapy. Genomic studies have identified mutations within the quinolone-resistance-determining regions (QRDRs) of the *gyrA* and *gyrB* genes, causing resistance against antibiotics such as ciprofloxacin and levofloxacin [56,59]. Mutations in topoisomerase IV (*parC* and *parE*) [59,60,61] further reduce fluoroquinolone binding affinity and contribute to elevated minimum inhibitory concentrations (MICs). As seen in *Elizabethkingia anophelis*, resistance-conferring mutations can accumulate rapidly under antibiotic selective pressure, resulting in substantial increases in MICs over short treatment durations [62].

Beyond these mutations, overexpression of efflux pumps and reduced outer membrane permeability due to porin under-expression further limit intracellular antibiotic accumulation [60,63]. Certain studies have also shown that when levofloxacin is used as a singular agent, MICs may increase rapidly with multiple QRDR mutations. Hence, it has been recommended as a combination therapy to delay the increase in MIC [60,64].

The interpretation of these findings is limited by substantial heterogeneity in testing methodologies, interpretive criteria and incomplete treatment data, particularly in outbreak settings where co-infection is common. Co-infections include other multidrug-resistant organisms, most commonly *Pseudomonas aeruginosa*, *Enterococcus faecium* and *Acinetobacter baumannii* [65]. Hence, there remains no consensus on first-line antimicrobial therapy that currently exists.

### 2.6. Laboratory Identification

Accurate species identification is necessary for therapeutic decision-making and epidemiological surveillance. Currently, biochemical-based phenotyping systems (such as VITEK 2 or Phoenix) and Matrix-Assisted Laser Desorption/Ionisation–Time-of-Flight Mass Spectrometry (MALDI-TOF MS) systems are widely used for routine microbial identification [29]. However, misidentification of species is well-documented.

Multiple studies have demonstrated that isolates initially identified as *E. meningoseptica* using automated phenotypic identification systems or earlier MALDI-TOF MS databases, were subsequently reclassified as *E. anopheles* [29,38,58,62] or *E. miricola* [63] following 16S rRNA sequencing or whole-genome sequencing [29,38,58,62]. In a retrospective analysis of 79 *Elizabethkingia* bloodstream isolates, Bruker MALDI Biotyper (bioMérieux)—a MALDI-TOF MS platform—identified 96.2% of samples as *E. meningoseptica* and 3.8% as *E. miricola*. However, almost full-length 16S rRNA sequencing revealed 98.7% of isolates to be *E. anophelis*, with a sole *E. meningoseptica* isolate [38]. Similarly, Lin et al. evaluated the performance of four commercial identification systems (API/ID32, Phoenix 100 ID/AST, Vitek 2 and Vitek MS) against 16S rRNA gene sequencing [39]. Its results revealed low concordance, with species-level identification accuracy ranging from only 24.5% to 26.5%.

Nonetheless, the use of 16S rRNA gene sequencing does have its limitations. Whole-genome sequencing studies have demonstrated that several closely related *Elizabethkingia* species—*E. bruuniana*, *E. miricola*, *E. ursingii* and *E. occulta*—share high 16S rRNA sequence identities (98.80–99.60%), rendering reliable species identification based on 16S rRNA difficult [51]. As such, whole-genome sequencing remains the most reliable method for definitive species identification, though its complexity, cost and time-consuming nature limit its routine clinical use [51]. Despite these constraints, 16S rRNA gene sequencing provides a higher taxonomic resolution than phenotypic methods and early MALDI-TOF MS with limited database coverage and remains a commonly used comparator in the absence of whole-genome sequencing.

Despite systematic misidentification of species by earlier default MALDI-TOF MS databases, recent studies have shown that they can reliably and rapidly identify clinically relevant *Elizabethkingia* species with expanded reference databases [51]. Additionally, a recent study done in 2025 by Mahapatra et al. showed that conventional PCR performed with specific primers targeting *E. anophelis* and *E. meningoseptica* demonstrated excellent concordance with MALDI-TOF MS, allowing for the identification of all clinical isolates as *E. anophelis* with 100% sensitivity and specificity. The development and use of species-specific PCR assays would hence aid in the rapid and accurate identification of *Elizabethkingia* spp. [38], especially in low-resource environments where MALDI-TOF MS and whole-genome sequencing are not routinely available [52].

Timely and accurate species-level identification of *Elizabethkingia* is critical for clinical management [51]. Empirical therapy for non-fermenting Gram-negative infections frequently includes antimicrobial agents to which *Elizabethkingia* spp. are intrinsically resistant. In a meta-analysis of 1000 patients identified to have *Elizabethkingia* infections, it was reported that the use of inappropriate antimicrobial therapy, even in empirical therapy, is associated with increased mortality [38]. Delayed recognition or non-specific identification results in prolonged exposure to ineffective empirical therapy, contributing to increased mortality, prolonged hospitalisation and adverse neurological outcomes, particularly among critically ill adults and neonates [2,54]. Enhanced diagnostic capacity, including the use of expanded MALDI-TOF MS databases, species-specific PCR 16S rRNA gene sequencing and whole-genome sequencing, is essential to support early targeted therapy, antimicrobial stewardship and effective infection control.

## 3. Literature Search Strategy

A comprehensive literature search was conducted using PubMed on 18 October 2025 to identify relevant studies on *Elizabethkingia* infections. Search terms included “*Elizabethkingia*”, “*Chryseobacterium meningosepticum*”, “*Chryseobacterium miricola*”, “*Flavobacterium meningosepticum*”, “*Flavobacterium miricola*”, “*Elizabethkingia anophelis*” and “*Elizabethkingia endophytica*”. Retrieved articles were screened for relevance, and case reports, case series and outbreak investigations related to human infections were included. In addition to full-text articles, abstracts containing sufficient relevant clinical information were also included where full texts were unavailable. Studies not relevant to human clinical infections, including purely environmental, animal or in vitro studies, were excluded. Only articles published in English were eligible for inclusion. A total of 300 case reports, 41 case series and 33 outbreak investigations were selected for analysis.

Patients aged ≥18 years were classified as adults, while those aged <18 years were classified as paediatric patients.

Species were analysed as reported in the original publications. Legacy nomenclature (e.g., *Chryseobacterium meningosepticum* and *Flavobacterium meningosepticum*) was not reclassified and was retained as separate categories to reflect historical reporting practices.

Generative artificial intelligence tools, specifically ChatGPT, were used to support aspects of data collection, analysis and interpretation. The authors remained actively involved in all study inclusion and exclusion decisions, data interpretation and manuscript preparation, and take full responsibility for verifying the accuracy of the information and the correctness of its interpretation throughout the research process.

## 4. Results

The full dataset is provided in the Supplementary Material.

### 4.1. Adult Population

#### 4.1.1. Case Reports

**Figure 1:**
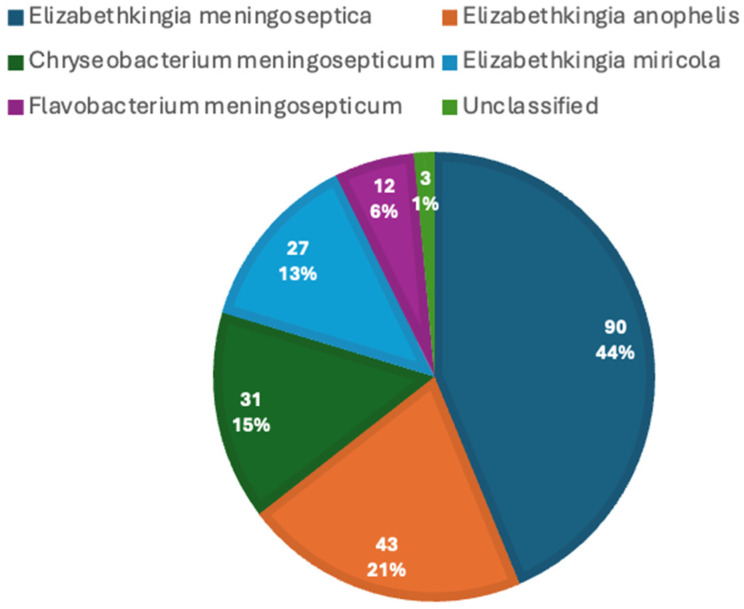
Distribution of *Elizabethkingia* species in adult case reports.

A total of 206 adult cases of *Elizabethkingia* were identified, spanning from 1969 to 2025 and originating from multiple geographic regions, with the highest representation from Asia (121/206, 58.7%). Figure 1 depicts the distribution of *Elizabethkingia* species in case reports, with the highest being *Elizabethkingia meningoseptica.*

Among adult case reports, infections predominantly affected older adults and were largely healthcare-associated, occurring in patients with a high burden of comorbidity. Bacteraemia, sepsis and pneumonia were the most common presentations, and mortality was substantial, particularly among patients with significant underlying diseases, as seen in Table 1. Healthcare exposures, such as the use of indwelling devices, prolonged hospitalisation and recent surgery, were also risk factors of developing *Elizabethkingia* infections [14,44,59].

#### 4.1.2. Case Series

A total of 31 adult case series were analysed, including a small number of mixed adult–paediatric studies (*n* = 4) in which age-disaggregated adult data could not be separately extracted. The included literature comprised predominantly retrospective case series, encompassing cohorts published between the early 1990s and 2024. Adult case series varied in cohort size from 11 to 127 patients. Pooled adult sex distribution revealed a male predominance of 834/1320 (63.2%). Reported mean ages across adult case series ranged from approximately 47 to 82 years, while reported median ages ranged from approximately 52 to 78.5 years, reflecting a predominantly middle-aged to elderly population.

**Figure 2:**
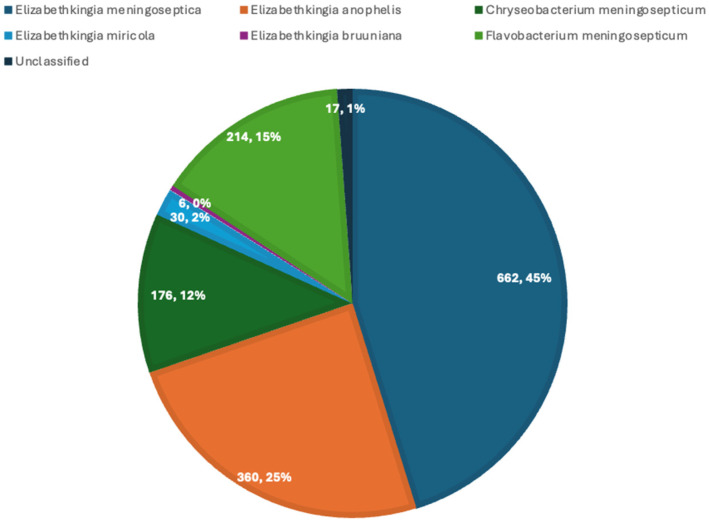
Distribution of *Elizabethkingia* species in adult case series.

Figure 2 depicts the distribution of *Elizabethkingia* species in the case series, with the highest being *Elizabethkingia meningoseptica.*

Hospital-acquired infection predominated, reported in the vast majority of adult cohorts (approximately 26–29 of 31 studies), typically accounting for approximately 70–95% of cases within individual series. These infections occurred most commonly among patients admitted to Intensive Care Units, those requiring mechanical ventilation and those with indwelling intravascular devices.

Adult patients exhibited a high burden of comorbidity, with underlying disease reported in nearly all adult case series, affecting between 60% and 100% of patients within individual cohorts. Across case series, the most frequently reported comorbidities include malignancy (approximately 6–45%), diabetes mellitus (approximately 11–63%), cardiovascular disease, including hypertension (approximately 19–77%), chronic renal disease or haemodialysis dependence, excluding wholly dialysis-dependent cohorts (approximately 5–33%), chronic lung disease (approximately 12–63%) and liver disease (approximately 5–30%). In one large adult cohort (*n* = 127), malignancy was present in 57 patients (45%), and prior antibiotic exposure was reported in 98 patients (77%) [57]. Immunosuppression, including chemotherapy, corticosteroid therapy or solid organ or stem cell transplantation, was reported in 20–45% in mixed and adult cohorts. Intensive Care Unit (ICU) admission, prolonged hospitalisation, mechanical ventilation and indwelling device use were common, with invasive device exposure reported in 50–90% of cases.

Bacteraemia or sepsis were the dominant clinical presentations, reported in approximately 24 of 31 (77.4%) adult case series. Pneumonia was the second most frequent manifestation, occurring in approximately 20 of 31 (64.5%) adult case series. Less common clinical presentations included urinary tract, intra-abdominal, biliary tract, soft tissue and central nervous system infections.

Adult patients generally experienced prolonged hospitalisation, with reported lengths of stay frequently exceeding 2–3 weeks, and in some ICU-based cohorts, extending beyond 30 days. Mortality was substantial, with in-hospital or 28-day mortality rates ranging from 2.9% (1/33) to over 50% [58]; in certain studies, in-hospital mortality reached 65.6%, with a 14-day mortality of 43.0% [28,66].

#### 4.1.3. Outbreaks

Among the literature sieved, 14 studies reporting outbreaks of *Elizabethkingia* spp. predominantly in the adult population were included, spanning the continents of Asia, Europe and North America. Countries with reported outbreaks include Taiwan, Singapore, India, the United States, the United Kingdom, France and Spain. Thirteen studies described hospital-based outbreaks, while one study described a statewide outbreak in Wisconsin; one of the hospital-based outbreaks represented a health-system subset of the Wisconsin outbreak. Excluding the health-system report, 12 hospital-based outbreaks were analysed by the clinical setting in which the outbreak occurred, as seen in Table 2.

Across these hospital-based outbreaks (excluding the statewide investigation), the number of patients affected ranged from 4 to 30, while the statewide Wisconsin outbreak involved 63 confirmed cases. Two studies included mixed-age cohorts. As adult cases were primarily reported, these studies were retained for analysis in this section. Excluding the mixed-age studies, older adults were disproportionately affected, with the median or mean age ranging from 45 to 82 years in studies that provided demographic data. In studies that reported sex distribution (6/14), male patients comprised 0 to 73.3% of cases.

Mechanical ventilation was the most consistently documented exposure, explicitly reported in 7 of 14 (50%) studies. Prior antibiotic exposure was reported in 4 of 12 (28.6%) studies with data on risk factors. Comorbidities were inconsistently reported across studies, while malignancy was reported in 4 (28.6%) studies and diabetes mellitus in 2 (14.3%), suggesting under-ascertainment due to heterogeneous reporting and extraction.

Clinical syndromes were recorded in 10 studies, and respiratory involvement was reported in all 10 (100%). This included pneumonia and tracheobronchitis. Notably, ventilator-associated pneumonia was documented in three studies. Colonisation without documented invasive disease was recorded in two studies (20%).

Crude mortality outcomes were highly variable across outbreaks, where mortality was reported in 11 of 14 studies (78.6%), ranging from 0% to 73.1%, with the highest mortality observed in the outbreak in a respiratory care centre (RCC) in Taiwan. This is in contrast to a mortality rate of 11.6% in the background the same RCC population without *Elizabethkingia* infection. Death attributable to *Elizabethkingia* infection was only documented in one study to be 18.2% [67].

*E. anophelis* was the most frequently reported organism, identified in 5 of 14 studies (35.7%), followed by *E. meningoseptica* in 4 studies (28.6%). *F. meningosepticum* was reported in 3 studies (21.4%), while *C. meningosepticum* and *E. miricola* were each reported in 1 study (7.1%). Detection methods were reported in 13 (92.9%) studies, with MALDI-TOF MS used in 6 (46.2%) of those specifying an identification method. Pulsed-field gel electrophoresis (PFGE) and whole-genome sequencing (WGS) were also used in a subset of investigations to support outbreak relatedness and confirm clonal transmission.

Environmental source identification was done as part of outbreak investigations, with a definitive reservoir identified in 6 of 14 (42.9%) studies. When identified, sources most commonly implicated water-associated reservoirs (5/14, 35.7%), including sinks, taps, aerators, heavily colonised water systems of a respiratory care unit (18/34 [52.9%] tap samples positive in Taiwan) and water-containing respiratory equipment (ventilator humidifier water). One outbreak detailed contaminated aqueous chlorhexidine solutions across two hospitals. In contrast, 7 of 14 studies (50%)—including the statewide Wisconsin outbreak and its health-system subset—reported failure to identify a source despite comprehensive investigation. One study (7.1%) described probable environmental contributions, without a definitive microbiologic linkage between environmental and clinical isolates, where correction of the equipment pasteurisation procedure resulted in prompt outbreak termination.

### 4.2. Paediatric Population

#### 4.2.1. Case Reports

**Figure 3:**
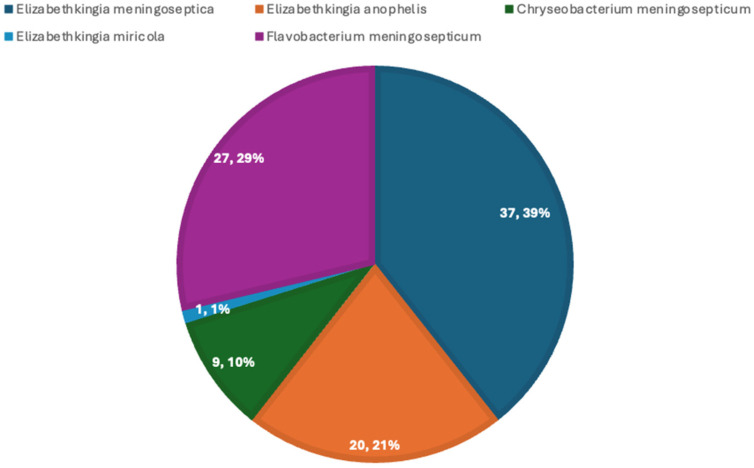
Distribution of *Elizabethkingia* species in paediatric case reports.

A total of 94 paediatric case reports were included in this review. *E. meningoseptica* was the most frequently reported species, as seen in Figure 3. All studies were published between 1966 and 2025 and originated from various regions of Asia, Europe, South America, Africa, Middle East and the United States of America, reflecting a global distribution.

Paediatric *Elizabethkingia* infections predominantly affected neonates and were largely hospital-acquired. Meningitis was the most frequent clinical presentation, and despite moderate overall mortality, neurological sequelae were common among survivors, particularly following meningitis, as seen in Table 3.

#### 4.2.2. Case Series

A total of 10 paediatric case series were included, primarily retrospective case series published between the 1980s and 2023. Among studies which reported the number of patients, each series varied in cohort size from 6 to 22. Sex distribution reported in 8 studies demonstrated a slight female predominance, with the pooled data available comprising 65.3% of female patients (47/72). Reported mean ages in two studies ranged from 13.25 days to 3.5 years, while median ages available in two studies ranged from 7 days to 1 year. Overall, ages ranged from 0 days to 17 years across the case series.

Acquisition status was reported in seven paediatric case series. Among these, infections were predominantly hospital-acquired (6/7, 85.7%), with one series reporting mixed hospital and community acquisition. No paediatric case series described exclusively community-acquired infection.

**Figure 4:**
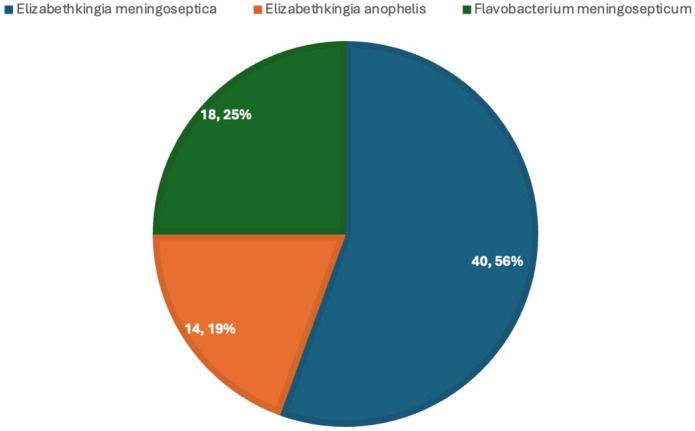
Distribution of *Elizabethkingia* species in paediatric case series.

Figure 4 depicts the distribution of *Elizabethkingia* species in case series, with the highest being *Elizabethkingia meningoseptica.*

Paediatric patients exhibited a high burden of baseline vulnerability and medical complexity. Across case series, the most frequently reported comorbidities include prematurity, which was reported in six studies, affecting approximately 30–86% of patients within individual cohorts. Early neonatal respiratory compromise was also documented, with respiratory distress at birth reported up to 100% in one study. Other risk factors include malignancy, which was reported in two studies, ranging from 46% to 55%, and congenital anomalies, which were noted in at least one series. Exposure to prior antibiotic use and central-venous catheter use were also cited as exposures in two studies.

Across the paediatric case series, central nervous system and bloodstream infection syndromes predominated, with meningitis, bacteraemia and sepsis most frequently reported. Meningitis was reported as a clinical syndrome in 8/10 (80%) case series, whereas pneumonia was reported in 2/10 (20%) series. Neurological complications were common. Several paediatric series described communicating hydrocephalus, seizures or long-term neurodevelopmental impairment among survivors of neonatal meningitis, with at least 3 out of the 11 cases reporting such complications [3].

Among the seven series that reported mortality, the crude mortality rate ranged from 15.4% (2/13) to 57.1% (4/7).

#### 4.2.3. Outbreaks

Among the literature reviewed, 19 outbreaks of *Elizabethkingia* spp. and related genera were included, spanning 11 countries across Asia, Europe, the Middle East, Africa and North and South America. Countries with reported outbreaks include India, Turkey, USA, Brazil, Greece, Denmark, Malaysia, Mauritius, Norway, Israel and Singapore. All (*n* = 19) studies were analysed by clinical setting in which the outbreaks occurred, as seen in Table 4.

Across the 19 paediatric outbreak studies, the predominant facility type was the neonatal intensive care unit (NICU). Thirteen studies (68.4%) reported outbreaks occurring exclusively in a NICU. One study (5.3%) described an outbreak spanning both the NICU and a paediatric ward, bringing the total number of studies involving NICU exposure to 14 (73.7%). Additional settings included paediatric ICU or children’s ICUs (2/19, 10.5%), neonatal wards (1/19, 5.3%), newborn nurseries (1/19, 5.3%) and general children’s wards (1/19, 5.3%). Overall, intensive care settings accounted for 16 of 19 outbreaks (84.2%).

Across these outbreaks, the number of paediatric patients affected ranged from 3 to 92. Age data were reported in 14 studies (73.7%), with median ages predominantly within the neonatal period. Three outbreaks involved patients beyond the neonatal period: one outbreak included five older paediatric patients [68], another had a small cohort of three patients ranging from 2.8 months to 4.8 years [69] and one involved 6 patients with an age range of 4 to 11 months [48].

Risk factor data were available in 12 of 19 studies (63.2%). Prematurity was reported in 7 of 12 studies (58.3%), with proportions ranging from 50.0% to 100% within individual cohorts. Low birth weight was reported in 9 of 12 studies (75.0%), affecting 18.4% to 100% of patients. Respiratory support prior to infection, including mechanical ventilation or non-invasive support, was reported in 3 of 12 studies (25.0%), with mechanical ventilation specifically documented in 2 studies. Prior exposure to broad-spectrum antibiotics was reported in 2 of 12 studies (16.7%).

Clinical syndromes were variably reported across studies. Meningitis was the most frequently documented diagnosis, explicitly reported in 11 of 19 studies (57.9%). Sepsis and bacteraemia were reported in 8 of 19 studies (42.1%). Colonisation without invasive disease was reported in 7 of 19 studies (36.8%).

Across studies reporting deaths, crude mortality rates ranged from 12.0% to 100%. High mortality (≥25%) was observed in 9 of 19 studies (47.4%), including three studies with mortality ≥50% and one study reporting 100% mortality (6/6). Neurological complications were prominent amongst infected patients, particularly in those with meningitis. Hydrocephalus and/or ventriculitis were reported in seven studies, with proportions ranging from 20.0% (1/5) to 50.0% (8/16 survivors). Ventriculoperitoneal shunt placement was required in several studies, and long-term sequelae included cerebral palsy, spasticity and mental retardation. An outbreak study of *E. meningoseptica* identified through a univariate analysis that shock at presentation was significantly associated with increased mortality (*p* = 0.04), while seizures (*p* = 0.04) and elevated cerebrospinal fluid protein levels (*p* = 0.01) at illness onset were predictive of progressive hydrocephalus among surviving neonates [70].

*F. meningosepticum* was the most frequently identified organism, reported in 7 of 19 studies (36.8%), followed by *C. meningosepticum* in 5 studies (26.3%). *E. meningoseptica* was reported in five studies (15.8%), including one mixed outbreak. Meanwhile, *E. anophelis* was reported in three studies (15.8%). One study described a mixed outbreak involving both *E. anophelis* and *E. meningoseptica*. Detection methods were reported in 18/19 (94.7%) studies, with culture-based methods predominating. Among the seven studies that specified an identification platform, MALDI-TOF MS was used in three (42.9%). Pulsed-field gel electrophoresis (PFGE) and arbitrary-primed PCR (AP-PCR) with antibiogram typing were used in a subset of investigations to support outbreak relatedness and demonstrate clonal transmission.

Environmental sampling identified a definitive outbreak source in 10 of 19 studies (52.6%). Among these, water-associated or moist environmental reservoirs were identified in six studies (31.6%), including tap outlets with aerators, sinks and basins, incubator and ventilator water, suction fluids and sink drains. Medical equipment or consumables were implicated in five studies (26.3%), including shared lipid stock bottles, nutritional solution, central venous catheter lines, ventilator device and milk bottle teats stored in water-filled containers. One study (5.3%) demonstrated person–environment transmission, with isolation of *Elizabethkingia* spp. from healthcare worker hands, as well as multiple high-touch surfaces. While one study did not manage to identify the source of the outbreak, outbreak control was achieved following decontamination of overhead water tanks.

### 4.3. Summary Table Comparing Adult and Paediatric Studies

Table 5 illustrates the comparison between all adult and paediatric case reports, case series and outbreak studies.

## 5. Discussion

### 5.1. Principal Findings from Results

This comprehensive review synthesises over five decades (1966–2025) of published literature on *Elizabethkingia* infections across adult and paediatric populations, integrating evidence from case reports, case series and outbreak investigations. Several consistent themes emerge: *Elizabethkingia* infections are predominantly healthcare-associated, occur most frequently in critically ill or highly vulnerable patients, demonstrate extensive intrinsic antimicrobial resistance and are associated with substantial mortality and long-term morbidity, particularly in neonates. Collectively, these findings reinforce *Elizabethkingia* spp. as opportunistic but clinically significant nosocomial pathogens whose impact is likely underestimated due to diagnostic limitations and heterogeneous reporting.

### 5.2. Antimicrobial Resistance and Therapeutic Implications

Across populations and study designs, a consistent finding was the extensive intrinsic multidrug resistance of *Elizabethkingia* spp., including frequent resistance to β-lactams and near-universal resistance to carbapenems, which severely limits the effectiveness of standard empiric regimens for nosocomial Gram-negative infections. Evidence from case series, case reports and outbreak investigations demonstrates marked inter-isolate and inter-study variability in susceptibility profiles, undermining the reliability of empirical therapy [47]. This unpredictability is further compounded by the absence of standardised interpretive breakpoints, complicating treatment decisions even when in vitro data are available.

Resistance to multiple first-line Gram-negative agents likely contributes to the high rates of inappropriate initial therapy reported in both adult and neonatal intensive care settings. Intrinsic resistance to carbapenems and colistin further distinguishes *Elizabethkingia* from other non-fermenting Gram-negative bacilli and eliminates commonly relied-upon salvage options [53].

Among available agents, minocycline demonstrated the most consistently preserved in vitro activity across adult and paediatric cohorts, supporting its role as a key therapeutic option when susceptibility is confirmed, although evidence of clinical superiority remains largely observational. In contrast, apparent activity of trimethoprim–sulfamethoxazole, piperacillin–tazobactam and fluoroquinolones was highly inconsistent across studies [71]. Fluoroquinolone susceptibility was particularly unstable, with several reports documenting rapid increases in minimum inhibitory concentrations during therapy, consistent with rapid resistance evolution under antimicrobial pressure and raising concern regarding fluoroquinolone monotherapy.

Interpretation of susceptibility to agents traditionally directed against Gram-positive organisms, including vancomycin, remains problematic. Early reports suggesting activity were contradicted by later studies demonstrating elevated MICs or universal resistance, likely reflecting methodological limitations and poor correlation with clinical efficacy [71]. Similar variability has been reported for linezolid, chloramphenicol and tigecycline, reinforcing the need for cautious, context-specific interpretation of susceptibility results [72].

Paediatric and adult *Elizabethkingia* infections differ in clinical presentation: paediatric patients, particularly neonates, more frequently present with meningitis, whereas adults more commonly present with pneumonia. These differences in clinical syndrome—and the extent to which antibiotics can achieve effective concentrations at the site of infection—may contribute to the apparent variation in commonly reported active agents across age groups. In cases of meningitis refractory to treatment, alternative routes of antibiotic administration exist. Intrathecal or intraventricular antibiotic administration can bypass the blood–brain barrier and increase concentrations of the drug in the cerebrospinal fluid. In contrast, pneumonia relies on systemic antibiotic therapy and pulmonary penetration, which may be variable. Vancomycin penetrates lung tissue poorly [49] but can be administered intraventricularly, potentially explaining why vancomycin is more frequently reported as part of effective regimens in paediatric meningitis-dominant cohorts. Additionally, vancomycin monotherapy has been reported to be ineffective in some adult cohorts [73]. Meanwhile, in paediatric cohorts, vancomycin is often administered as part of combination therapy, such as with ciprofloxacin or rifampicin, rather than as monotherapy [15], and several neonatal case series have described successful clinical outcomes with vancomycin-containing regimens [74].

In vitro susceptibility to vancomycin is inconsistent, with some studies reporting high minimum inhibitory concentrations (MICs), showing that its apparent clinical efficacy may reflect treatment context rather than intrinsic antimicrobial activity alone [71].

Notably, early outbreak investigations also documented the rapid emergence of resistance during therapy, including the development of resistance to sulfisoxazole, erythromycin and vancomycin during treatment in a 1975 outbreak [75] and the emergence of high rifampicin MICs within days of therapy in a neonatal outbreak reported in 1984. Together, these findings underscore the central importance of early species-level identification, isolate-specific susceptibility testing and antimicrobial stewardship in the management of *Elizabethkingia* infections, as well as the need for prospective studies to define optimal treatment strategies and combination regimens.

### 5.3. Insights from Outbreak Investigations and Clinical Implications

Outbreak investigations can provide important clinical insights besides epidemiology. In a 2024 case-control outbreak study of neonatal *E. anophelis* bloodstream infection [76], apnoea emerged as the most common presenting symptom, which occurred more often among *E. anophelis* cases than among neonates with other culture-positive bloodstream infections (57.9% vs. 15.8%). Interestingly, central venous catheter use appeared more strongly associated with non-*E. anophelis* bloodstream infections, suggesting that *E. anophelis* transmission may be less driven by in-dwelling devices. Univariate analysis also revealed prematurity and requirement for inotropic support to be significantly associated with mortality. Notably, *E. anophelis* infection was reported to occur even in otherwise healthy neonates, often those on enteral feeds and lacking traditional neonatal sepsis risk factors. This suggests that the absence of recognised host risk factors does not preclude infection. Hence, there is a great need to minimise environmental reservoirs in the hospital.

Older outbreak reports similarly clarify clinically relevant patterns that may be overlooked when focusing only on invasive disease. In the 1975 United States outbreak of *F. meningosepticum* colonisation, the upper respiratory tract was colonised first in most infants, suggesting a predilection for this anatomical site [75]. Importantly, antibiotic therapy directed at colonisation did not confer benefit in that report; infants who received no specific therapy had a shorter mean duration of colonisation (9.3 days) than those treated with antibiotics (22.7 days). This finding cautions against reflexive antimicrobial treatment for colonisation and highlights the potential for antibiotics to prolong carriage, either through the effect of selective pressure or the failure to eradicate the underlying reservoir.

Across both adult and paediatric settings, outbreak investigations consistently characterise *Elizabethkingia* spp. as environmentally persistent, water-associated healthcare pathogens that are difficult to eradicate with standard infection-control measures. Intervention findings across outbreaks further suggest that conventional strategies may be inadequate due to biofilm production. In one critical care unit outbreak with heavy sink and tap colonisation, daily chlorination did not eliminate *E. meningoseptica*, and post–hand-washing alcohol-based hand gel was also ineffective; by contrast, automated high-pressure flushing of clinical taps achieved eradication, implying that mechanical disruption of biofilms may be more effective than chemical disinfection alone [10]. In a ventilator-associated outbreak, autoclaving contaminated humidifier boxes was required to terminate transmission, reinforcing the need for proper equipment sterilisation when reusable water-containing components are implicated [77].

Successful outbreak control appears to rely on multimodal strategies in addition to conventional infection-control practices. This includes targeted water-system interventions (e.g., changing sink taps, removing tap aerators) and sterilisation of equipment, rather than relying on routine chemical disinfection alone. Collectively, these data highlight the limitations of conventional approaches against *Elizabethkingia*.

### 5.4. Limitations

Despite the breadth of literature included, this review presents several limitations. Most included studies were retrospective and observational, with substantial heterogeneity in study design, reporting standards and laboratory methodologies, especially in the identification of *Elizabethkingia* spp. Antimicrobial susceptibility testing methods also varied widely, and the absence of standardised interpretive breakpoints limits direct comparison across studies. Historical changes in taxonomy and diagnostic platforms likely resulted in species misclassification, particularly in earlier reports. The variation of taxonomic classifications over time also further limited comparability.

Furthermore, patient-level data were often incomplete, with missing data on age, clinical presentation, comorbidities, antimicrobial susceptibilities and therapy, as well as long-term outcomes.

Nevertheless, the consistency of key findings across populations, settings and decades using case reports, case series and outbreak investigations can support the robustness of the main findings of this review.

## 6. Conclusions

*Elizabethkingia* spp. are emerging healthcare-associated pathogens that disproportionately affect critically ill adults with extensive healthcare exposure and neonates. Adult infections most commonly present as bacteraemia or pneumonia, whereas paediatric disease is dominated by neonatal meningitis, frequently complicated by severe neurological sequelae. Across populations, infections are characterised by extensive intrinsic antimicrobial resistance, rendering standard empirical Gram-negative regimens ineffective and contributing to delayed appropriate therapy.

Although agents such as minocycline, trimethoprim-sulfamethoxazole and fluoroquinolones have demonstrated relative in vitro activity, susceptibility patterns are heterogeneous, and resistance may emerge during therapy, underscoring the necessity of isolate-specific, susceptibility-guided treatment. Ongoing limitations in species-level identification, particularly historical misclassification of *E. anophelis* as *E. meningoseptica*, have further obscured epidemiological trends and may have influenced reported outcomes.

Outbreak investigations highlight the organism’s environmental persistence and biofilm-forming capacity, with evidence that mechanical disruption and water-system engineering interventions may be more effective than chemical disinfection alone. Accurate and timely species-level identification, targeted antimicrobial therapy and rigorous water-focused infection prevention strategies are therefore essential to reduce the morbidity and mortality associated with *Elizabethkingia* infections.

## Figures and Tables

**Table 1:** Clinical characteristics and outcomes of adult case reports (*n* = 206).

Demographics	Age, mean (range)	56.97 years (18–104 years)	203/206 reported with data
	Male sex	116 cases (61.7%)	188/206 reported with data
	Female sex	72 cases (38.3%)	
Acquisition	Healthcare-associated	115 cases (72.3%)	159/206 reported with data
	Community-associated	44 cases (27.7%)	
Risk factors	At least 1 comorbidity	185 cases (89.8%)	206 cases all reported with data
	Diabetes mellitus	47 cases (22.8%)	
	Chronic kidney disease	39 cases (18.9%)	
Clinical presentation	Bacteraemia/sepsis	84 cases (42.0%)	200/206 reported with data
	Pneumonia	36 cases (18.0%)	
	Ventilator-associated pneumonia	5 cases (2.5%)	
	Meningitis	23 cases (11.5%)	
	Skin & soft tissue infection	13 cases (6.5%)	
	Endocarditis/pericarditis	7 cases (3.5%)	
	Ocular infection	6 cases (3.0%)	
Outcome	Mortality	61 cases (32.8%)	186/206 reported with data

**Table 2:** Distribution of hospital-based *Elizabethkingia* spp. outbreaks by clinical setting (*n* = 14).

Clinical setting	ICU/Critical Care Unit	7 studies (58.3%)	12/14 reported with data
	Respiratory care centre	1 study (8.3%)	
	Long-term acute care hospital	1 study (8.3%)	
	Infectious diseases unit	1 study (8.3%)	
	Mixed cardiac/medical/chronic ventilation units	1 study (8.3%)	
	General hospital wards	1 study (8.3%)	

**Table 3:** Clinical characteristics and outcomes of paediatric case reports (*n* = 94).

Demographics	Age range	Newborn–17 years	
	Mean age	1.57 years	
	Neonates (≤28 days)	61 cases	
	Male sex	45 (54.2%)	83/94 reported with data
	Female sex	38 (45.8%)	
Acquisition	Hospital-acquired infection	51 (73.9%)	69/94 reported with data
	Community-acquired infection	18 (26.1%)	
Comorbidities	Prematurity	30 (44.8%)	67/94 reported with data
	No comorbidities	27 (28.7%)	
Clinical presentation	Meningitis	61 (65.6%)	93/94 reported with data
	Sepsis/bacteraemia	44 (47.3%)	
	Pneumonia	11 (11.8%)	
	Other focal infections	VAP, SSTI, keratitis	
Outcomes	Mortality	21 (23.3%)	90/94 reported with data
	Neurological sequelae (survivors)	15 (16.7%)	
	Type of sequelae	Hydrocephalus, developmental delay, seizures	
	Long-term follow-up	Inconsistently reported	

**Table 4:** Distribution of hospital-based *Elizabethkingia* spp. outbreaks by clinical setting (*n* = 19).

Clinical Setting	Number of Studies (*n* = 19)
NICU only	13 studies (68.4%)
NICU and paediatric ward	1 study (5.3%)
Total studies involving NICU exposure	14 studies (73.7%)
Paediatric or children’s ICU	2 studies (10.5%)
Total studies involving intensive care settings	16 studies (84.2%)
Neonatal ward	1 study (5.3%)
Newborn nursery	1 study (5.3%)
General children’s ward	1 study (5.3%)

**Table 5:** Summary table of all adult and paediatric case reports, case series and outbreak studies.

Variable	Adults	Paediatric/Neonates
Number of patients	*n* = 1946	*n* = 422
Mean age (range)/years *	56.97 (18–104)	1.57 (0–17)
Male sex *	61.70%	54.22%
Common species	*E. anophelis*, *E. meningoseptica*	*E. meningoseptica*, *F. meningosepticum*, *E. anophelis*
Common diagnoses	Bacteraemia/sepsis, pneumonia	Bacteraemia/sepsis, meningitis
Key comorbidities	Malignancy/Immunosuppression, Diabetes Mellitus, CKD, ICU stay, indwelling medical devices	Prematurity, low birth weight, NICU admission, indwelling medical devices, congenital abnormalities
Common complications	Mostly deaths	Hydrocephalus, developmental delay, seizures
Antibiotic susceptibility	Minocycline, TMP-SMX, fluoroquinolones, pip-tazo, rifampicin	Minocycline, TMP-SMX, fluoroquinolones, pip-tazo, vancomycin
Antibiotic resistance	Carbapenems, Cephalosporins, Aminoglycosides, Colistin, Vancomycin	Carbapenems, Aminoglycosides, Colistin, Cephalosporins
Mortality *	32.80%	23.30%

* Statistics derived from case reports. TMP-SMX: trimethoprim/sulfamethoxazole, pip-tazo: piperacillin-tazobactam.

## Data Availability

The original contributions presented in this study are included in the article/Supplementary Material. Further inquiries can be directed to the corresponding author.

## Supplementary Materials

The following supporting information can be downloaded at: https://www.mdpi.com/article/10.3390/pathogens15030278/s1, Table S1: Results Table.
